# Molecular Dynamics and Free Energy Calculations Predict Binding Mode and Affinity Determinants of Specialized Pro-Resolving Mediators at GPR101

**DOI:** 10.1021/acsomega.5c13583

**Published:** 2026-07-12

**Authors:** Daniel Haga Hasselstrøm, Majd Awad, Trond Vidar Hansen, Osman Gani

**Affiliations:** Department of Pharmacy, Section for Pharmaceutical Chemistry, University of Oslo, 0316 Oslo, Norway

## Abstract

GPR101 is an orphan G protein-coupled receptor (GPCR) with unusually high constitutive activity and has recently emerged as a target for specialized pro-resolving mediators (SPMs), endogenous lipids that actively terminate inflammation and promote tissue repair. Given the therapeutic relevance of pro-resolution signaling in chronic pain and inflammatory disorders, understanding how SPMs engage GPR101 is of great significance. Although cryo-EM structures suggest an occluded orthosteric cavity, SPMs such as RvD5_n‑3 DPA_ are potent agonists, creating uncertainty about their binding modes. Long-timescale molecular dynamics (MD) simulations, MM-GBSA per-residue energy decomposition, residue-interaction network analysis, and alchemical relative binding free-energy (RBFE) calculations were used to predict interactions between SPMs with GPR101. MD trajectories revealed a stable RvD5_n‑3 DPA_ pose beneath an extracellular loop, stabilized by M184, W186, and Y415. Transmembrane distance metrics across five independent 1 μs MD simulation trajectories showed persistent stabilization of an active-like state even without modeled G-protein coupling. RBFE analyses quantified the scaffold-dependent effects of C17 alcohol stereochemistry, oxidation, methylation, and 3-oxa substitution. A double mutant cycle calculation identified a coupling between C17 alcohol and residue M184. Novel dual-modified analogs were computationally predicted to retain high affinity while improving metabolic stability. Benchmarking demonstrated that membrane-free thermodynamic integration (AMBER) yielded accurate, low-variance results with shorter wall time than membrane-inclusive replica exchange (NAMD). These results provide the first atomistic model of SPM binding to GPR101 and establish an RBFE-guided framework for designing next-generation pro-resolving mediator analogs with enhanced pro-resolving effects and stability.

## Graphical abstract

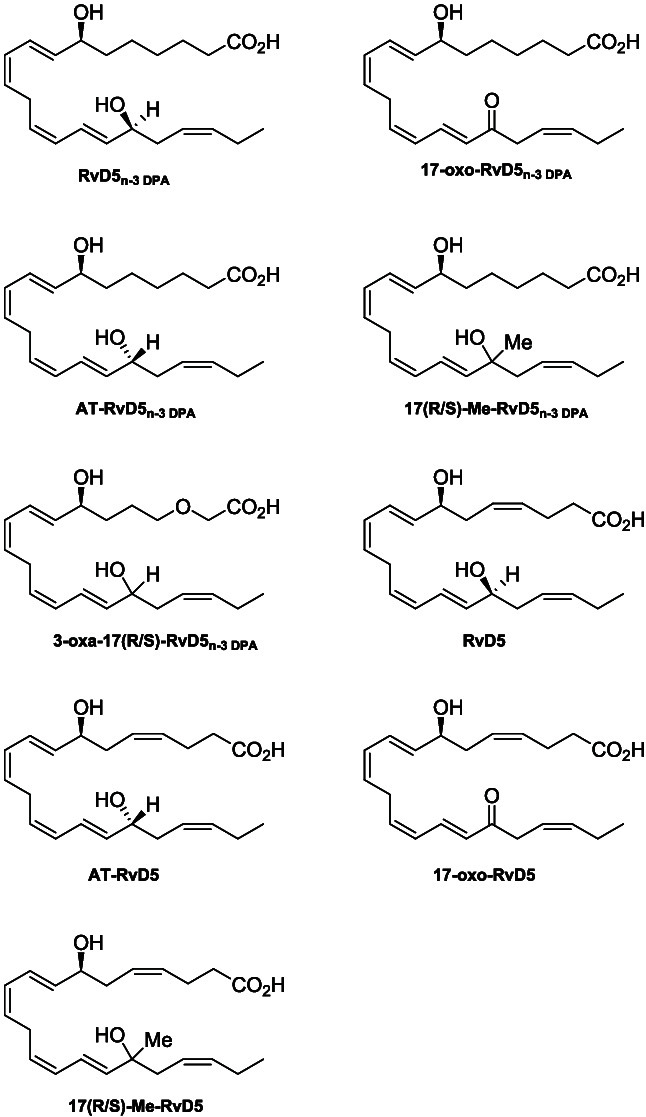

## 1 Introduction

The resolution of inflammation is a coordinated process that is essential for maintaining health. When this process fails, inflammation can become chronic, contributing to the development of various human diseases.^1^ Advances in our understanding of how acute inflammation resolves have revealed new classes of endogenous compounds, including the lipoxins, resolvins, protectins, and maresins, collectively known as specialized pro-resolving mediators (SPMs), which actively and potently terminate inflammation.^2^ Although identified in inflammation resolution, SPMs are conserved mediators involved in host defense, pain modulation, organ protection, and tissue remodeling.^3^ The resolution phase involves tightly regulated cellular and biochemical processes that restore homeostasis^4^ where SPMs are biosynthesized locally via lipoxygenase and cyclooxygenase enzymes using omega-3 (ω-3) and omega-6 (ω-6) polyunsaturated fatty acids as substrates. The bioactions by SPMs are largely mediated through potent and stereoselective agonism of specific G-protein-coupled receptors (GPCRs).^5,6^ Due to their endogenous origin and potent bioactivity, SPMs represent promising lead compounds in resolution pharmacology^3^ and for the development of novel anti-inflammatory therapeutics.^7^

RvD5_n‑3 DPA_, an SPM derived from n-3 docosapentaenoic acid (n-3 DPA)^8,9^ is regulated in a diurnal manner in human peripheral blood.^10^ Notably, patients with cardiovascular disease exhibit reduced levels of RvD5_n‑3 DPA_, which correlates with increased activation of peripheral blood neutrophils, monocytes, and platelets. This SPM has also been shown to exert tissue-protective effects by regulating leukocyte trafficking and responses in murine models. Additionally, RvD5_n‑3 DPA_ modulates intestinal epithelial barrier function. Reduced levels of this oxygenated product during inflammatory arthritis are associated with increased intestinal permeability and joint inflammation.^11^ Moreover, RvD5_n‑3 DPA_ was found to bind and activate the orphan receptor GPR101, with a calculated EC_50_ value of 4.6 × 10^–12^ M.^12^ GPR101 is expressed on human peripheral blood leukocytes, and its functional importance was demonstrated in GPR101-deficient mice, which failed to exhibit the protective effects of RvD5_n‑3 DPA_ in models of inflammatory arthritis and infectious inflammation.^12^ Further studies using macrophages lacking GPR101 revealed the receptor’s critical role in regulating macrophage biology, mediated in part by RvD5_n‑3 DPA_.^12^ Disruption of GPR101 signaling resulted in heightened and prolonged inflammatory responses, highlighting its role in the timely resolution of inflammation.^13^ The metabolic inactivation of RvD5_n‑3 DPA_ has also been investigated,^11^ where incubation of the mediator in the presence of 15-prostaglandin dehydrogenase (15-PGDH) afforded the oxidized product, 17-oxo-RvD_5n‑3 DPA_, (Scheme 1). Unlike its parent SPM, the oxidized metabolite exhibited no bioactivity in macrophage assays. Additional experiments revealed that both mRNA and protein levels of 15-PGDH were upregulated in the small intestine of arthritic mice. Furthermore, immune complexes (antigen–antibody complexes) were shown to increase 15-PGDH expression in macrophages.^11^

**Scheme 1:**
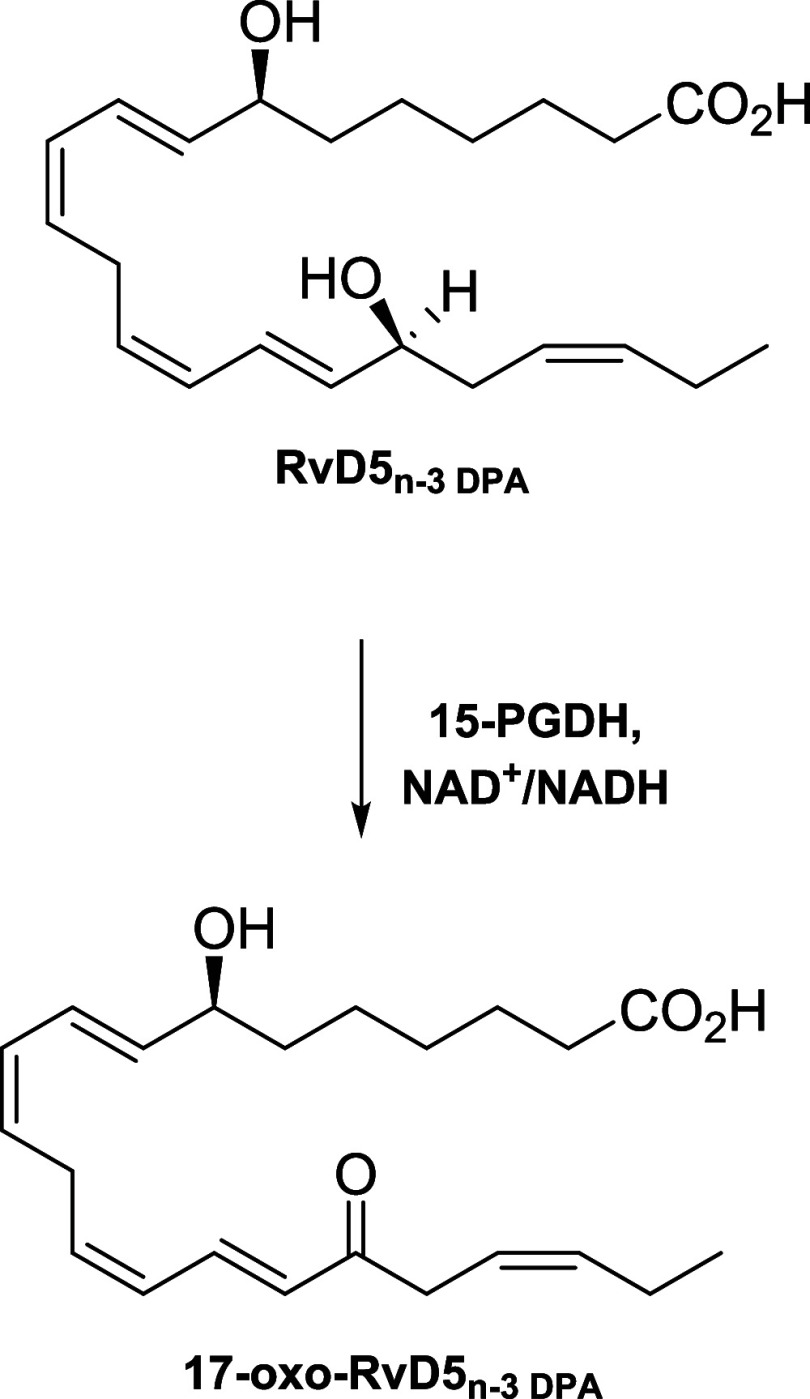
Metabolic Inactivation of SPM RvD_5n‑3 DPA_ by 15-Prostaglandin Dehydrogenase (15-PGDH)^a^

a Oxidation of the 17-hydroxy group affords the corresponding α,β-unsaturated ketone, 17-oxo-RvD5_n‑3 DPA_. Drawn using *ChemDraw* (PerkinElmer Informatics, Version 20.1).

Despite current evidence highlighting the importance of RvD5_n‑3 DPA_ and its metabolic products in GPR101 signaling and resolution biology, the molecular basis of agonist recognition by GPR101 remains poorly understood. To address this gap, molecular dynamics (MD) simulations and relative binding free energy (RBFE) calculations were employed to examine RvD5_n‑3 DPA_, its structural analogs and metabolites. Analyses of these compounds provide insights into the binding mode of SPMs, structural ligand determinants of GPR101 affinity, and emerging structure–activity relationships for this class of SPMs.

## 2 Results and Discussion

GPR101 is an orphan GPCR that was recently identified as a target of specialized pro-resolving mediators, which are endogenous lipids that orchestrate the resolution of inflammation. Recently published cryo-EM structures have suggested that the orthosteric site may be partly occluded, raising questions about whether agonists can be accommodated.^14^ This uncertainty is compounded by the fact that SPMs are chemically sensitive, making direct structural determination of SPM–GPCR complexes especially difficult.^15^ As a result, computational approaches are indispensable for defining how these ligands engage their receptors.

Much of the current computational insight into SPM–GPCR interactions has come from the work of Nunes et al., who systematically applied MD simulations, residue interaction analysis, end point free-energy methods such as MM-GBSA/MM-PBSA, and metrics such as the percentage of active-state conformations during trajectories as a proxy for agonist potency. Multiple studies have investigated pro-resolving and pro-inflammatory mediators at FPR2 ^16–18^ and BLT1.^19^ These studies provide valuable qualitative and semiquantitative perspectives on receptor activation and ligand recognition, laying an important foundation for the field. Building on this groundwork, the present study integrates five replicates of 1 μs molecular dynamics (MD) simulations, residue-level interaction quantification, and rigorous alchemical relative binding free-energy (RBFE) calculations to elucidate how specialized SPMs engage the GPR101 receptor and benchmark the performance of two complementary RBFE protocols. The 12 ligands examined comprised native SPMs, primary metabolites, and metabolically stabilized analogs, as shown in Figure 1, which provides an overview of the chemical series analyzed in this study.

**Figure 1:**
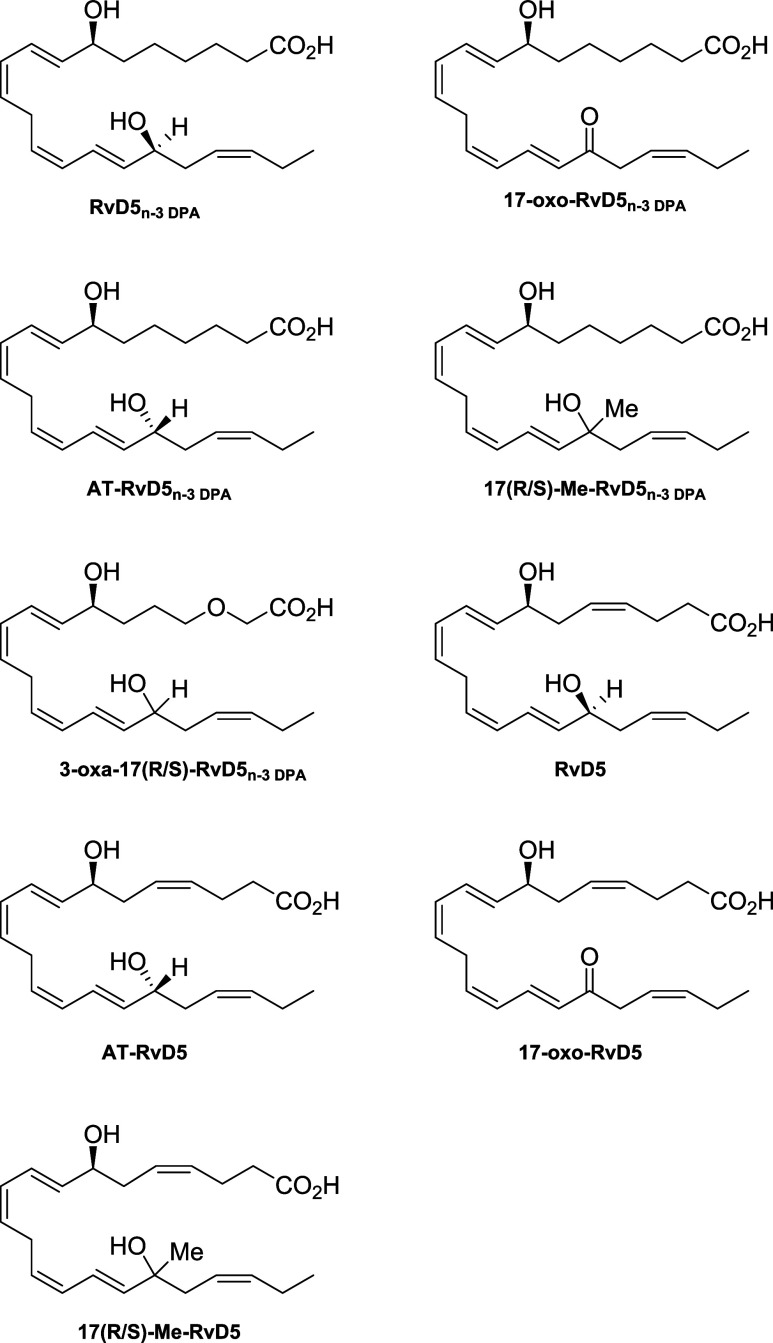
2D chemical structures and names of the ligand set used for modeling in this study, drawn using *ChemDraw* (PerkinElmer Informatics, Version 20.1). Epimeric mixtures are shown where applicable; individual stereoisomers were modeled separately.

### 2.1 Binding Mode of RvD5_n‑3 DPA_ in GPR101

RvD5_n‑3 DPA_ remained stable within the orthosteric binding pocket of GPR101 throughout all five simulations, as indicated by an average protein backbone RMSF of 1.33 Å for residues within the transmembrane helices (Table S1) and a ligand RMSD of 1.90 Å (Table S2 and Figure S1). Notably, the highest ligand fluctuation was observed at the partially extracellular exposed carboxylate group and the nearby segment of the alkyl chain (corresponding to carbons 1–6). Both metrics showed an early convergence. The ligand RMSD reached approximately 1.99 Å after three runs and changed by only 0.09 Å when the fourth and fifth runs were added, corresponding to 95% convergence. The protein TM-backbone RMSF stabilized at approximately 1.32 Å after three runs, with only a 0.02 Å change after adding the final two runs.

In contrast, two 300 ns MD simulation runs of RvD5_n‑3 DPA_ at the allosteric membrane site, corresponding to the ligand AA-14 position in the reference PDB structure, gave a TM-backbone RMSF of 1.00 ± 0.73 Å and a ligand RMSD of 10.12 ± 2.44 Å. The large and unstable ligand displacement suggests that this site does not represent a stable or physiologically relevant binding mode for RvD5_n‑3 DPA_ and similar SPMs.

To identify stable and probable binding modes of RvD5_n‑3 DPA_ in GPR101, the trajectories from the five independent orthosteric site MD simulations were clustered based on the ligand RMSD.

Figure 2 shows the overlaid ligand poses from five simulations. The extracellular-exposed carboxylate headgroup displayed the greatest flexibility, whereas the hydrophobic tail was stabilized by consistent insertion beneath ECL2 into the canonical orthosteric pocket.

**Figure 2:**
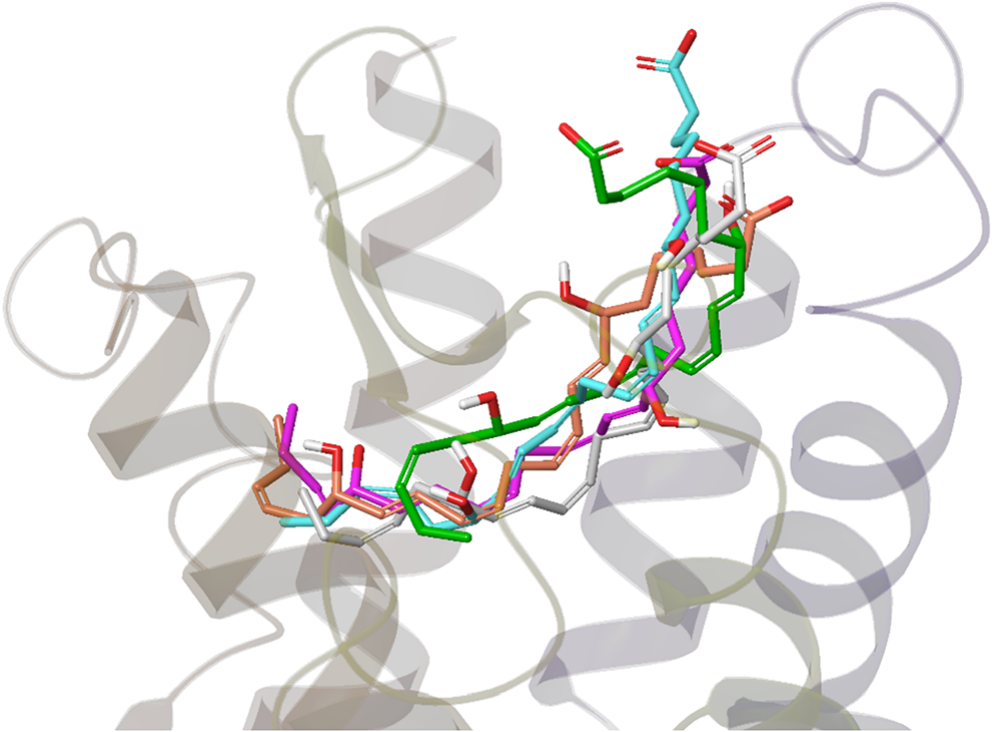
Five representative binding poses of RvD5_n‑3 DPA_ in GPR101 derived from k-means clustering of trajectories from five independent 1-μs MD simulations. Pre-MD docking was performed in the orthosteric binding site. The hydrophobic tail of RvD5_n‑3 DPA_ was consistently and stably inserted beneath ECL2 in the canonical orthosteric cavity, whereas the carboxylate headgroup remained more flexible and solvent-exposed during the simulations.

One representative stable pose was selected for analysis from these ensembles. In this pose, the ligand’s carboxylate group formed electrostatic interactions with R178 and the backbone of E428. The C7 hydroxy establishes a direct hydrogen bond with the W186 side chain and a water-mediated hydrogen bond with Y415. Near the ligand ω tail, the C17 alcohol engages in a direct hydrogen bond with the C182 backbone carbonyl, in addition to water-mediated hydrogen bonds with the backbones of Y415 and M184 (Figure 3).

**Figure 3:**
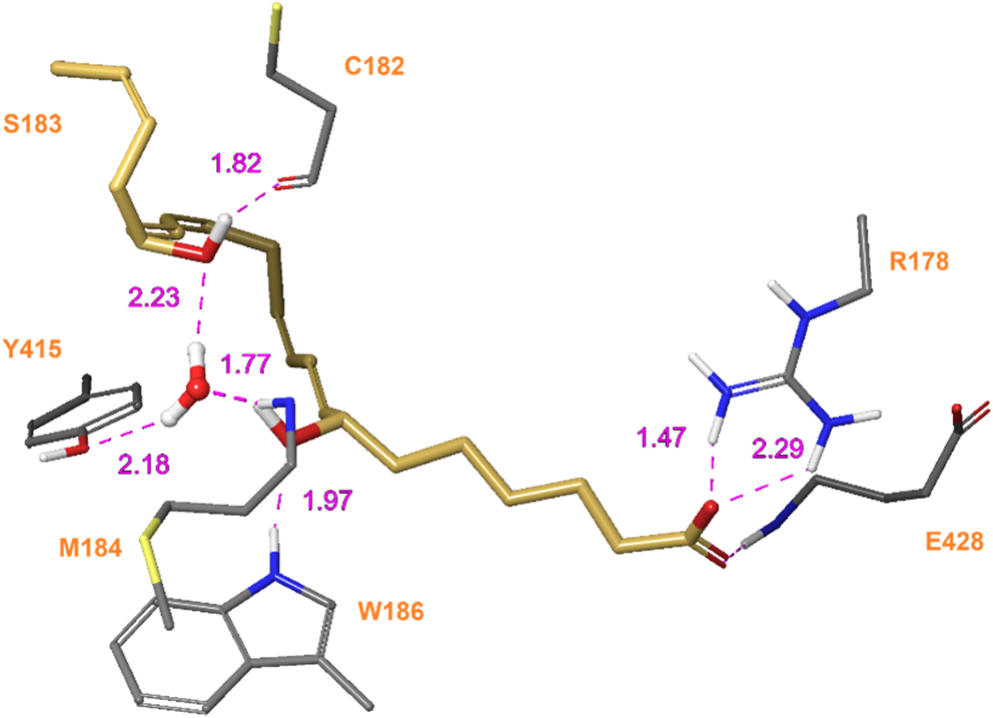
Polar interactions between RvD5_n‑3 DPA_ (yellow) and GPR101 residues. Water-mediated contacts, hydrogen bonds and electrostatic interactions are shown as dashed purple lines with distances (Å).

### 2.2 Residue Quantification Analysis

Quantification of interactions from the molecular dynamics simulations of the specialized pro-resolving mediator RvD5_n‑3 DPA_ bound to the orthosteric site of GPR101 provided detailed insights into ligand–receptor interactions, revealing distinct patterns of engagement involving specific ligand moieties and receptor residues distributed across several transmembrane (TM) segments (Table S3 and Figure 4).

**Figure 4:**
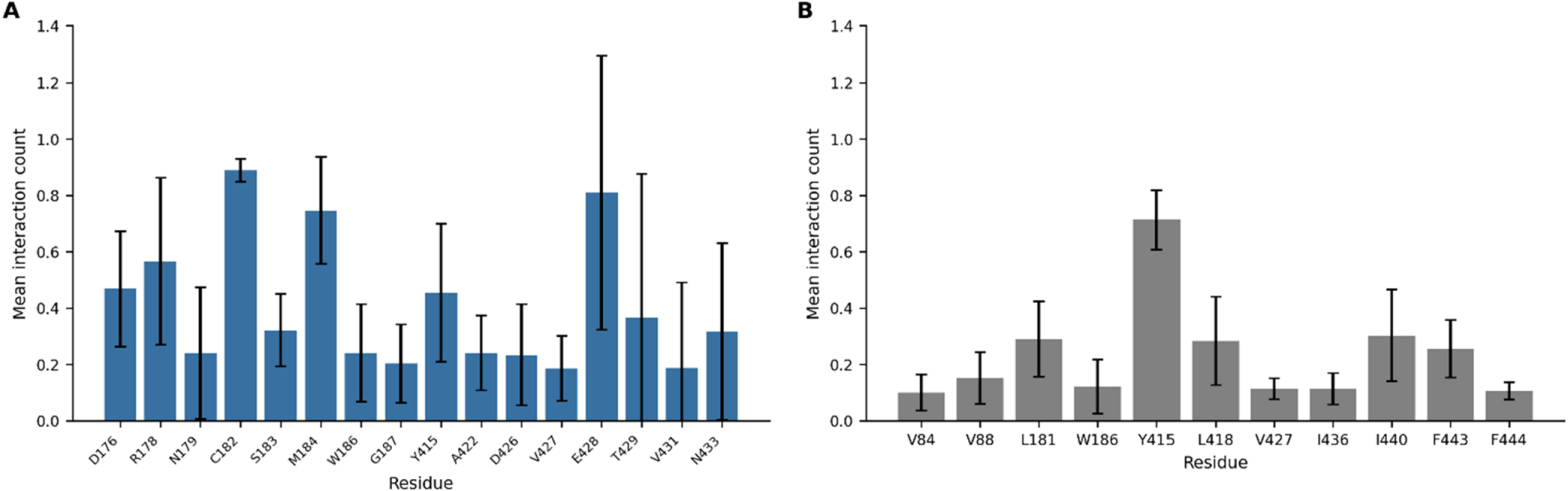
Bar chart of mean interaction counts between RvD5_n‑3 DPA_ and GPR101 residues from five independent runs of 1 μs MD simulations, generated using the Desmond Simulation Interactions Diagram (SID) module. Polar interactions include hydrogen bonds, ionic interactions, and water bridges; nonpolar interactions include hydrophobic contacts, π–π stacking, and π–cation interactions. Only residues exhibiting a mean interaction frequency >10% across simulations are shown. Panel (A) (blue) shows the polar interactions, and panel (B) (gray) shows the nonpolar interactions. The error bars represent the standard deviation across the simulations.

The flexible carboxylate moiety of RvD5_n‑3 DPA_ engaged with several residues located at the extracellularly exposed part of the binding site, in ECL2, TM1, TM6, and TM7. Specific interactions involved R34 (TM1), R178 and N179 (ECL2), A422, D426 (via water-mediated interaction), V427, E428 (water-mediated), T429, V431, and Q433 (TM6 and TM7). Notably, R178 and E428 exhibited particularly high-frequency interactions, suggesting their critical roles in anchoring the ligand’s carboxylate group.

The C7 alcohol of RvD5_n‑3 DPA_ interacted with residues D176, R178, N179, S183, and M184 from ECL2. RvD5_n‑3 DPA_’s C17 alcohol moiety formed stable, high-frequency contacts predominantly with residues M184 and C182 (ECL2).

Furthermore, the extensive hydrophobic interactions provided by residues V84, W87, V88 (TM2), L181 (ECL2), Y415 (TM6), F443, F444 (TM7), W186 (ECL2), L418, V427 (TM6), I436, I439, and I440 (TM7) formed a hydrophobic environment surrounding the ligand’s hydrocarbon chain during the simulations.

Importantly, three of the residues most frequently contacted by the ligand; M184, W186, and Y415 have previously been identified by mutagenesis as critical for GPR101 function.^14^ The agreement between simulation-predicted interactions and experimental loss-of-function mutations provides strong support for the predicted binding mode, suggesting that the MD-derived pose captures crucial features of how SPMs engage the receptor.

Additionally, residue Y415 corresponds to position 6.51 in Ballesteros–Weinstein numbering (GPCRdb), a position that frequently forms part of the orthosteric ligand-binding pocket in class A GPCRs. Aromatic residues at position 6.51 are known to contribute to affinity and efficacy in the β_2_-adrenergic and muscarinic M_2_ receptors.^20,21^ The persistent involvement of Y415 observed in our simulations is therefore consistent with conserved structural roles of position 6.51 in stabilizing ligand engagement within class A GPCR orthosteric sites.

Residues M184 and W186 are located within ECL2 immediately downstream of the conserved cysteine (C182) that forms the canonical TM3–ECL2 disulfide bond characteristic of class A GPCRs. Residues in this region is known to contribute to ligand binding and receptor activation in many GPCRs.^22^ The strong and persistent contacts with M184 and W186 observed in the present simulations are consistent with the established functional importance of the ECL2–TM bundle interface in GPCR ligand recognition and activation.

In summary, these results provide the first atomistic predicted model of SPM binding at GPR101 and resolve the uncertainty raised by cryo-EM structures suggesting steric occupation of the orthosteric site. The simulations suggest that SPMs can adopt a semicanonical orthosteric binding mode beneath the ECL2, where they reinforce receptor activation by stabilizing the active-state conformation. In this model, ECL2–TM bundle contacts contribute to basal signaling, while SPM binding provides an additional layer of stabilization. This dual mechanism may explain how SPMs enhance GPR101 activity despite the apparent structural constraints of this pocket.

### 2.3 Receptor Activation

The TM3-TM6 distance, a structural hallmark of GPCR activation,^23^ was used to assess whether GPR101 in complex with RvD5_n‑3 DPA_ maintained an active-like conformation during five 1 μs MD simulations in the absence of a coupling partner. Across the five independent 1 μs trajectories, the mean TM3-TM6 distance was 11.56 ± 0.44 Å, with 25.8 ± 16.3% of the frames exceeding the 12.21 Å threshold observed in the active-state structure from PDB: 8W8S (Table S4, Figures S2 and S3). This indicates that RvD5_n‑3 DPA_ stabilizes GPR101 close to the active-state geometry even without G-protein engagement. Although some intertrajectory variability was observed, the narrow spread in the mean TM3–TM6 distance indicates that the active-like conformation was consistently maintained. The broader variation in the percentage of open frames likely reflects transient fluctuations rather than true inactive or intermediate states, as well as the heuristic nature of the 12.21 Å cutoff derived from a single active-state structure.

Similar analyses of other GPCR systems show broad variability depending on receptor type and simulation setup. For example, Nunes et al. reported that RvD1 maintained FPR2 in an active-like state for approximately 75% of the simulation time, based on a 10.5 Å threshold derived from the experimental active structure.^16^ In contrast, a 10 μs MD simulation of the β10 μs MD simulation of the β_2_-adrenergic receptor with an agonist showed less than 10% of frames above its respective active-state threshold.^24^ Such differences highlight the system dependence of this metric. While the TM3-TM6 distance serves as a useful first indicator of receptor activation, a comprehensive understanding of GPR101 dynamics will require future accelerated MD^25^ and free-energy landscape analyses that also consider NPxxY motif rearrangements, sodium site occupancy, and internal water network dynamics, which are all known to play central roles in GPCR activation.^21,26,27^

### 2.4 MM-GBSA and Per-Residue Decomposition

Three independent 50 ns MD simulations of the RvD5-GPR101 complex were performed in a heterogeneous membrane environment using AMBER to estimate the overall binding strength and identify key stabilizing residues via MM-GBSA analysis. The computed Δ*G*_bind_ of −59.4 ± 0.7 kcal/mol indicates strong and stable ligand–receptor binding. Per-residue energy decomposition (Figure 5 and Table S5) revealed a substantial overlap with the high-frequency contact residues observed in the long-time scale Desmond simulations. Notably, key residues M184, W186, and Y415 contributed to the top 20 most favorable interactions. Solvent-exposed residues such as R178 and E428 presented negligible contributions to computed binding affinity, despite forming frequent contacts during MD simulations (Figure 4), likely because favorable electrostatic contacts with the carboxylate group of RvD5 are largely offset by desolvation penalties and solvent screening effects, yielding a minimal net contribution to the binding free-energy.

**Figure 5:**
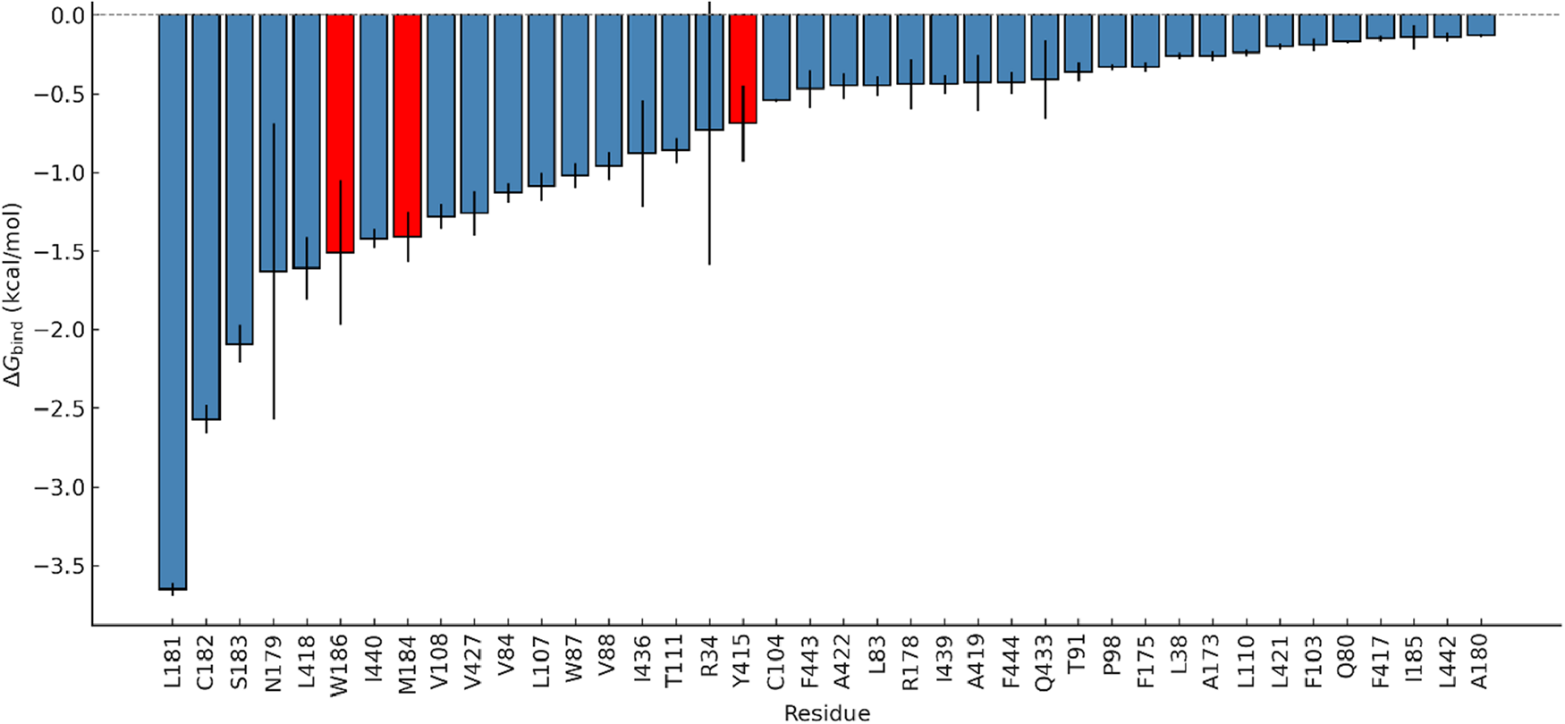
Per-residue MM-GBSA binding energy decomposition for GPR101 bound to RvD5_n‑3 DPA_. The bars show the mean Δ*G*_bind_ contribution for each residue (kcal/mol), with negative values indicating favorable interactions. Residues M184, W186, and Y415 (red) exhibit strong stabilizing contributions and were also identified as key contact residues in the long-time scale MD analyses and thermodynamic-integration calculations, consistent with their previously reported importance for receptor activation in mutagenesis studies.

### 2.5 Free Energy Protocols: AMBER vs NAMD

A comparison of relative binding free energy predictions obtained from the AMBER TI and NAMD FEP/λ-REMD protocols (Tables 1 and 2) showed no significant correlation in ligand rank ordering (Spearman’s ρ = 0.03, *p* = 0.91). The two approaches differed on average by an RMSE of 2.26 kcal/mol, and while many transformations yielded comparable results, strong outliers such as 3-oxa-17­(R)-Me-RvD5_n‑3 DPA_ and RvD5 contributed disproportionately to the overall error (Figure S4).

**Table 1:** Binding Free Energy Differences (ΔΔ*G* ± SEM) for Each Ligand Relative to the Reference RvD5_n‑3 DPA_, as Calculated by AMBER TI and NAMD FEP/λ-REMD, Expressed in kcal/mol

ligand	AMBER TI ΔΔ*G* ± SEM (kcal/mol)	NAMD FEP/λ-REMD ΔΔ*G* ± SEM (kcal/mol)
17-oxo-RvD5_n‑3 DPA_	–1.38 ± 0.13	–1.05 ± 0.30
AT-RvD5_n‑3 DPA_	–0.26 ± 0.06	+1.01 ± 0.36
17(S)-Me-RvD5_n‑3 DPA_	–0.67 ± 0.17	–0.82 ± 0.23
17(R)-Me-RvD5_n‑3 DPA_	+0.53 ± 0.14	–3.29 ± 0.61
3-oxa-17(S)-RvD5_n‑3 DPA_	+0.72 ± 0.48	+0.83 ± 0.36
3-oxa-17(R)-RvD5_n‑3 DPA_	+1.03 ± 0.31	+0.61 ± 0.22
3-oxa-17(S)-Me-RvD5_n‑3 DPA_	+0.22 ± 0.38	+0.89 ± 0.26
3-oxa-17(R)-Me-RvD5_n‑3 DPA_	+1.38 ± 0.42	+5.27 ± 0.47
RvD5	+0.19 ± 0.14	–3.06 ± 0.60
17-oxo-RvD5	+0.36 ± 0.40	+0.21 ± 0.31
AT-RvD5	+0.71 ± 0.47	–2.01 ± 0.32
17(S)-Me-RvD5	+1.17 ± 0.49	–2.40 ± 0.41
17(R)-Me-RvD5	+1.29 ± 0.43	–2.91 ± 0.43

**Table 2:** Binding Free Energy Differences (ΔΔ*G* ± SEM) for Ligands Relative to Reference RvD5, as Calculated by AMBER Thermodynamic Integration, Expressed in kcal/mol

ligand	AMBER TI ΔΔ*G* ± SEM (kcal/mol)
RvD5_n‑3 DPA_	–0.29 ± 0.15
AT-RvD5	+0.12 ± 0.03
17-oxo-RvD5	+1.25 ± 0.18
17(S)-Me-RvD5	+0.40 ± 0.19
17(R)-Me-RvD5	+2.11 ± 0.30

Benchmarking against β-arrestin recruitment assays yielded an experimental ΔΔ*G* of −1.67 kcal/mol for the transformation of RvD5_n‑3 DPA_ to RvD5. AMBER TI predicted +0.19 ± 0.14 kcal/mol (forward) and −0.29 ± 0.15 kcal/mol (reverse), corresponding to absolute errors of ∼1.9 kcal/mol although with excellent internal consistency and a small cycle closure error of +0.10 kcal/mol (Figure 6). In contrast, NAMD predicted a value −3.06 ± 0.60 kcal/mol. Although numerically closer to the EC_50_-derived estimate, this value carried a large uncertainty and produced an implausibly favorable shift relative to the broader structure–activity relationship (SAR) trend. Importantly, EC_50_-derived values are only an indirect proxy for the binding affinity in GPCRs, where receptor signaling amplification can uncouple functional efficacy from thermodynamic binding free energies. While EC_50_ values are not true measures of binding affinity in GPCRs, a recent benchmark showed that they can perform comparably to affinity data for RBFE validation, with an average RMSE of ∼1.5 kcal/mol across targets.^28^ In this context, the ∼1.9 kcal/mol error observed here falls within the expected performance range, which supports the reliability of the AMBER TI protocol for both retrospective analysis and prospective ligand design.

**Figure 6:**
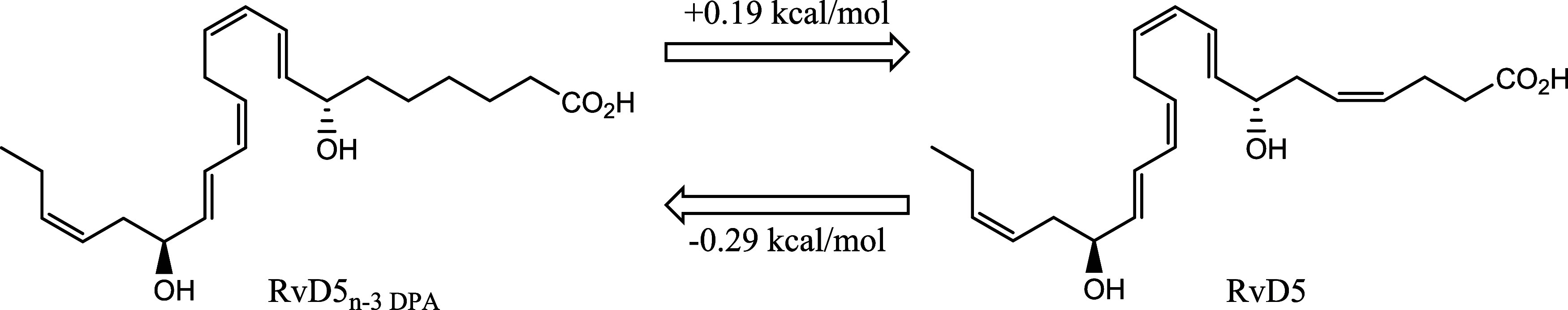
Binding free energy differences (ΔΔ*G*) for alchemical transformations between RvD5_n‑3 DPA_ and RvD5, computed using AMBER Thermodynamic Integration. The calculated cycle closure error was +0.10 kcal/mol.

For RvD5 metabolites and analogs, alchemical transformations using RvD5_n‑3 DPA_ as the reference ligand yielded relative binding free energies with relatively large SEMs compared to other transformations. It was suspected that ligand transformations involving multiple structural and stereochemical changes contributed to the sampling difficulties and thus lower precision of the calculated ΔΔ*G* values. To investigate this, the same four compounds were resimulated with AMBER TI using RvD5 as the reference ligand, and the alchemical transformations exhibited significantly smaller SEMs, indicating an improved precision. Importantly, only the absolute magnitudes of the RBFE values shifted, whereas the overall SAR trend was preserved regardless of the chosen reference ligand. Cycle closures were between 0.4 and 1.1 kcal/mol (Figures S5–S8), consistent with acceptable thermodynamic soundness given the large conformational space accessible to these flexible ligands, and supportive of the overall robustness of the AMBER protocol for this system.

The AMBER TI data set revealed chemically intuitive SAR features, such as the additive and predictable effects of hydroxy stereochemistry and C17 substitution. In contrast, the NAMD λ-REMD protocol occasionally produced less systematic and, in some cases, implausible affinity shifts, undermining confidence in its predictive value for this system. Conceptually, NAMD has the advantage of being membrane-inclusive and utilizing enhanced sampling, which should in principle capture important aspects of receptor–ligand binding for GPCRs. However, a recent benchmark has shown that membrane-free protocols often perform as well as membrane-inclusive approaches for membrane proteins, provided lipid bilayer interactions are not the primary determinants of ligand binding.^29^ In the present system, AMBER’s simplified protocol yielded tighter error estimates, likely because the absence of explicit membrane lipids reduced noise in the calculations, and possibly due to differences in sampling strategy between the two protocols. The AMBER protocol also required substantially less simulation time while still producing chemically reasonable SAR trends.

Overall, AMBER TI provided the most reliable and chemically interpretable results for this system. The NAMD values are reported in full for transparency, but the subsequent discussion focuses on the AMBER results, in line with the best practice recommendations to prioritize the most converged and interpretable protocol. This conclusion is system-dependent: for GPCRs where membrane-driven conformational states or lipid contacts are central to ligand recognition, membrane-inclusive protocols remain essential. However, for prospective ligand design in GPR101, AMBER TI represents a more pragmatic and robust choice.

### 2.6 17-Position Alcohol Stereochemistry

Relative binding free energy calculations using AMBER TI were performed for RvD5_n‑3 DPA_, RvD5, and their aspirin-triggered 17­(R)-epimers (Table 1), using RvD5_n‑3 DPA_ as the reference ligand. Transformations converged well, with SEMs < 0.5 kcal/mol even for more challenging cases.

For RvD5_n‑3 DPA_, the aspirin-triggered 17­(R)-epimer was predicted to maintain comparable, or slightly enhanced, affinity relative to the native S-epimer, indicating that the GPR101 pocket is permissive to both stereoisomers. In contrast, RvD5 displayed a modest stereochemical preference: initial results suggested a ∼0.5 kcal/mol penalty for the 17­(R)-epimer, but this estimate carried relatively large uncertainty. When recalculated with RvD5 as the reference ligand, the SEM decreased substantially and the predicted penalty was reduced to only +0.12 kcal/mol, suggesting near-equivalent binding. The one-step transformation of RvD5_n‑3 DPA_ to AT-RvD5 transformation overestimated this penalty, consistent with a small cycle closure error (−0.40 kcal/mol; Figure S5).

Overall, these results indicate that the stereochemistry of the C17 alcohol has a negligible impact on GPR101 binding affinity, within the precision limits of the AMBER TI calculations. While RvD5_n‑3 DPA_ appears fully permissive, and RvD5 shows only a nominal bias toward the 17­(S)-epimer, the predicted differences fall within the expected uncertainty of the RBFE calculations. This aligns with experimental observations of many different SPMs, where both 17-epimers often remain biologically active.^30^ Simultaneously, stereochemistry strongly influences metabolic stability, with 17­(S)-epimers more prone to oxidative metabolism by 15-PGDH and 17­(R)-epimers generally more persistent against this metabolic route and thus longer acting *in vivo*.^31^ Free energy calculations therefore provide a useful framework for designing SPM analogs that combine robust receptor affinity with improved metabolic resilience.

### 2.7 Double Mutant Cycle Analysis of the C17 Alcohol Hydrogen Bond

Residue quantification analysis and MM-GBSA per-residue decomposition suggested that M184 forms a stable hydrogen bond with the C17 alcohol group of RvD5_n‑3 DPA_, which significantly contributes to the binding affinity of this SPM. To further cross-validate and quantify the energetic importance of this interaction, a double mutant cycle was constructed in which RvD5_n‑3 DPA_ was alchemically transformed into its inactive 17-oxo analog in both the wild-type receptor and M184A mutant.

Thermodynamic integration yielded a transformation free energy of +1.25 ± 0.18 kcal/mol for the wild-type receptor and +0.33 ± 0.15 kcal/mol for the M184A mutant, corresponding to a coupling free energy change of −0.92 kcal/mol. This negative coupling term indicates that the C17 alcohol-M184 hydrogen bond provides a stabilizing contribution of approximately 1 kcal/mol to ligand binding. This confirms that the C17 alcohol group of RvD5_n‑3 DPA_ forms a specific and energetically meaningful hydrogen bond with M184, consistent with the per-residue decomposition analysis, and contributes to our understanding of the ability of this SPM to bind to the active state of GPR101.

### 2.8 SPM 17-Oxo Metabolites

For RvD5_n‑3 DPA_, the 17-oxo metabolite was predicted to bind GPR101 approximately an order of magnitude more strongly than the parent SPM. This suggests that a hydrogen-bond acceptor at C17, in combination with an appropriate ligand conformation, can maintain or even enhance the affinity.

In contrast, the 17-oxo metabolite of RvD5 showed modest effects. Using RvD5_n‑3 DPA_ as the reference ligand, the one-step transformation predicted retained affinity with only a small positive shift (+0.17 kcal/mol), although accompanied by a relatively large SEM. To reduce uncertainty, the transformation was repeated using RvD5 as the reference ligand, which yielded a substantially smaller SEM and indicated that the 17-oxo metabolite binds approximately eight times weaker than RvD5. Thus, while the absolute magnitudes varied depending on the reference ligand choice, the recalculated results confirmed the broader SAR trend: oxidation at C17 has opposite effects on the binding affinity depending on the SPM scaffold.

Metabolically, specialized pro-resolving mediators are inactivated by enzymes such as 15-prostaglandin dehydrogenase (15-PGDH), which oxidizes the C17 hydroxy group to an α,β-unsaturated ketone. These electrophilic metabolites can act as Michael acceptors and rapidly conjugate with glutathione or related nucleophiles. This may explain why, despite their predicted ability to bind GPR101, 17-oxo metabolites are consistently reported as biologically inactive *in vivo* across SPM classes. This discrepancy suggests that their lack of activity reflects metabolic instability, rather than poor receptor recognition. Looking forward, it may be valuable to explore SPM mimetics bearing nonconjugatable ketone functionalities, which could preserve favorable protein–ligand interactions at GPR101 while resisting rapid inactivation.

### 2.9 Metabolically Stabilized Analogs: C17-Methylation and 3-Oxa Substitution

Two established structural modifications known to improve pharmacokinetic stability were assessed for their impact on binding affinity. These modifications involve the formation of a tertiary alcohol at C17 through methylation and the inhibition of β-oxidation by 3-oxa substitution.

For RvD5_n‑3 DPA_, free energy calculations predicted that the 17­(S)-methyl epimer binds ∼3-fold more strongly than the parent compound, whereas the 17­(R)-epimer binding was favorable. A diastereomeric mixture of the two epimers is expected to exhibit an overall binding profile similar to that of RvD5_n‑3 DPA_ itself, but with enhanced metabolic resilience and potentially longer biological half-life. This prediction is consistent with recent *in vivo* findings by Ervik et al., who reported the superior analgesic and potency of the epimeric mixture compared to RvD5_n‑3 DPA_. Notably, the significant analgesic effects of these SPMs and their analogs were observed only in male mice.

In contrast, methylated analogs of RvD5 displayed weaker predicted binding than RvD5. Initial transformations, using RvD5_n‑3 DPA_ as the reference ligand, indicated affinity losses of +0.98 and +1.10 kcal/mol for the 17­(S)- and 17­(R)-epimers, respectively, although these results were accompanied by relatively large SEMs. The likely cause was the one-step transformation, which simultaneously reduced the C4–C5 double bond, introduced a methyl group, and inverted the alcohol stereochemistry. These substantial, simultaneous perturbations create sampling and convergence difficulties. Repeating the transformations with RvD5 as the reference ligand reduced SEMs and yielded more reliable estimates, again indicating that both 17-methyl analogs bind less favorably than RvD5. However, the 17­(S) epimer was predicted to be only approximately 2-fold weaker, whereas the 17­(R) epimer was approximately 31-fold weaker.

For 3-oxa analogs, the introduction of an ether moiety at C3 led to an approximate 3–5-fold reduction in the predicted binding affinity of both epimers relative to RvD5_n‑3 DPA_. This loss likely reflects both weaker hydrophobic contacts around the C3 region, but also conformational changes introduced by the ether linkage. These features may reduce the conformational adaptability of the polyunsaturated chain and alter the optimal positioning of distal functional groups within the binding pocket. Despite this binding penalty, prior studies show that 3-oxa substitution can substantially enhance metabolic stability and *in vivo* activity. 3-oxa-n-3 DPA has previously been prepared by total synthesis, and enzymology studies confirmed that it retained activity as a substrate for COX-2 and multiple LOX isoforms, comparable to native n-3 DPA. The resulting monohydroxylated products are structural analogs of endogenous SPM biosynthetic intermediates.^32^ In addition, 3-oxa-PD1_n‑3 DPA_ has been shown to produce potent analgesic and antipruritic effects in mouse models at low doses.^33,34^ Therefore, modest losses in receptor affinity may be offset by improved pharmacokinetic resilience, making 3-oxa substitution a pragmatic strategy.

Overall, C17 methylation seems to have scaffold-dependent effects, enhancing binding in RvD5_n‑3 DPA_ but reducing it in RvD5, whereas 3-oxa substitution consistently lowers affinity. These chemotype-specific outcomes emphasize the need to consider scaffold context when pursuing metabolic stabilization strategies and demonstrate how RBFE calculations can guide the rational design of SPM analogs by balancing receptor engagement with pharmacokinetic resilience.

### 2.10 Prospective RBFE: Dual-Modified Analogs

With the SAR patterns for individual modifications established, novel dual-modified analogs of RvD5_n‑3 DPA_ combining C17 methylation with 3-oxa substitution were evaluated. Thermodynamic integration revealed additive modification effects and a clear stereochemical dependence. The 17­(*R*)-methyl epimer showed a ∼10-fold reduction in binding affinity relative to the parent SPM, whereas the 3-oxa-17­(S)-Me-RvD5_n‑3 DPA_ analog retained strong predicted binding, nearly matching that of RvD5_n‑3 DPA_ (Figure 7). Notably, a diastereomeric mixture of these dual-modified analogs is predicted to yield only a ∼3–4-fold reduction in affinity overall, while offering the advantage of improved metabolic stability and, thus the potential for prolonged *in vivo* pharmacological effects.

**Figure 7:**
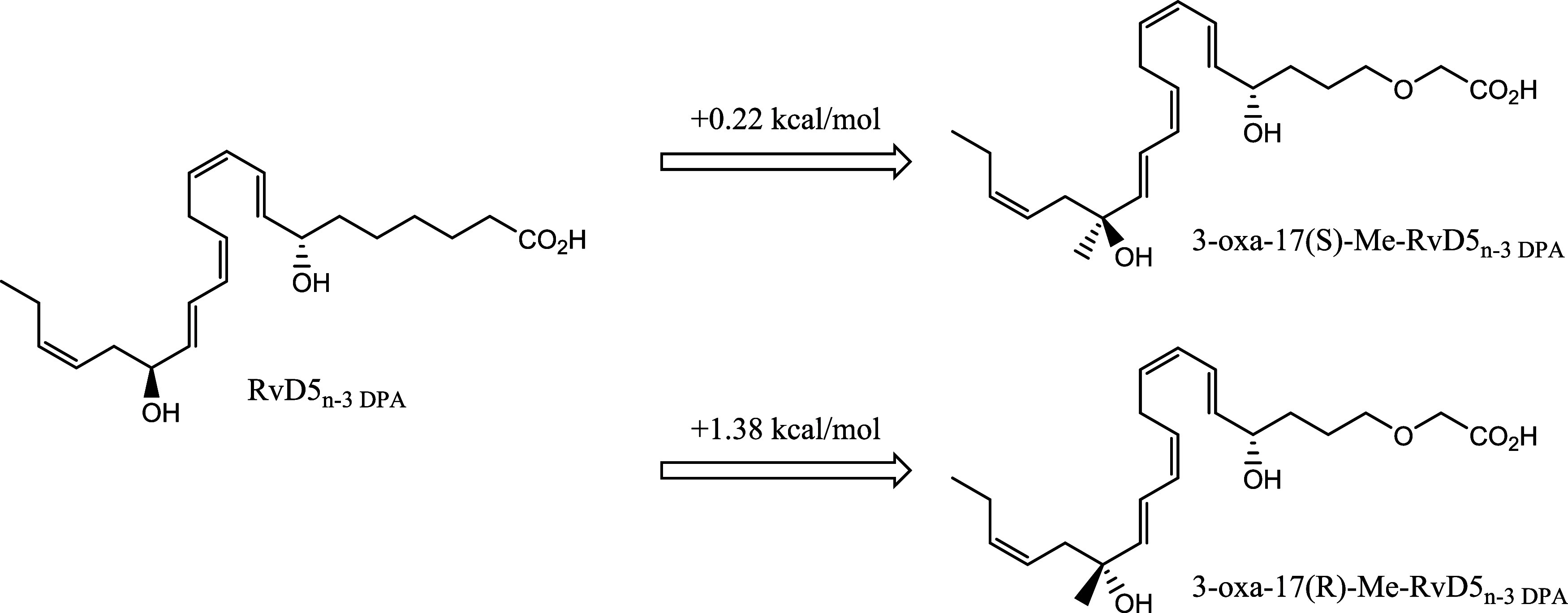
Binding free energy differences (ΔΔ*G*) for alchemical transformations between RvD5_n‑3 DPA_ and both epimers of the dual-modified analogs, computed using AMBER Thermodynamic Integration.

## 3 Conclusion

We present the first atomistic model of RvD5 and RvD5_n‑3 DPA_, as well as other SPM analogs binding to the active state of GPR101. Long-time scale molecular dynamics simulations predicted a semicanonical orthosteric binding mode beneath ECL2, supported by persistent contacts with residues previously validated by mutagenesis. These findings counter the view from cryo-EM structures that the orthosteric site is inaccessible and provide a structural rationale for GPR101 activation by SPMs.

Relative binding free-energy calculations quantified the scaffold-dependent effects of stereochemistry, oxidation, and metabolic stabilization. C17 methylation, 3-oxa substitution, and their combination modulated affinity in predictable ways, which can guide rational analog design. Benchmarking further showed that the membrane-free AMBER TI protocol achieved reliable accuracy with lower variance and wall time than the membrane-inclusive λ-REMD, supporting its use for prospective SPM design.

Overall, these results advance the molecular basis of SPM recognition by GPR101 and highlight the value of RBFE-guided design for optimizing both pharmacodynamic and pharmacokinetic properties. As the emerging field of resolution pharmacology continues to grow, computational approaches such as those developed here will be essential for guiding drug design and discovery, as well as the synthesis and testing of next generation of metabolically stabilized analogs with improved binding affinities. Current efforts are focused on the total synthesis and experimental validation of the predicted high-affinity dual-modified candidates.

## 4 Methods

### 4.1 Structure Preparation and Molecular Docking

The cryo-EM structure of active state GPR101 was retrieved from the PDB (ID: 8W8S) and the protein transmembrane core was extracted and prepared using the standard Protein Preparation Workflow protocol^35^ at pH 7.4. Ligands were prepared using the standard LigPrep protocol (**Schrödinger Release 2025–1**: LigPrep, Schrödinger, LLC, New York, NY, 2025.). Only the deprotonated states of the carboxylic acids were retained in the calculations. Molecular docking was performed using Glide Standard Precision (SP),^36,37^ with a grid centered around residues in the canonical orthosteric GPCR binding site underneath the ECL2. Procedures were carried out in Maestro (Schrödinger Release 2025–1).

### 4.2 Desmond Molecular Dynamics Simulation

The orientation of proteins in membranes server^38^ (PPM 2.0) was used for positioning of the lipid bilayer, and the docked complex of RvD5_n‑3 DPA_ was prepared for molecular dynamics simulations in Desmond,^39^ embedded in a homogeneous POPC bilayer, solvated with TIP3P water model, charge neutralized, added NaCl to 0.15 M and parametrized with the OPLS4 force field.^40^ Following the standard Desmond relaxation protocol, production runs of 300–1000 ns of all-atom molecular dynamics simulations in the NPT ensemble were performed at 310K (Nosé-Hoover chain thermostat) and 1 atm (Martyna-Tobias-Klein barostat), with frames written every 100 ps.

### 4.3 Protein–Ligand Residue Interaction Analyses

Maestro’s Simulation Interaction Diagram module was used for classification and quantification of polar and hydrophobic interactions between RvD5_n‑3 DPA_ and GPR101 from a total of 50 000 frames from the 5 independent 1 μs MD simulations.

Hydrogen bonds were defined using a donor–hydrogen–acceptor geometry with H···A distance <2.8 Å, D–H···A angle >120°, and H···A–X angle >90°. Water bridges were defined as hydrogen-bonded contacts mediated by a bridging water molecule, using H···A distance <2.7 Å, D–H···A angle >110°, and H···A–X angle >80° for the hydrogen bonds to the bridging water. Ionic interactions were defined between oppositely charged atoms within 3.7 Å. Hydrophobic contacts were defined as hydrophobic side chains within 3.6 Å of ligand aromatic or aliphatic carbon atoms. π–π stacking interactions were defined between aromatic rings as face-to-face stacking (centroid–centroid distance <4.4 Å and interplanar angle <30°) or edge-to-face (centroid–centroid distance <5.5 Å and interplanar angle >60°). π–cation interactions were defined when aromatic and charged group centroids were within 4.5 Å. Polar interaction counts were computed as the sum of hydrogen bonds, ionic interactions, and water bridges; nonpolar interaction counts were computed as the sum of hydrophobic contacts, π–π stacking, and π–cation interactions.

### 4.4 TM3-TM6 Distance Analysis

The relative displacement between transmembrane helices 3 and 6 (TM3-TM6) was quantified as a structural marker of GPR101 activation. Distances were measured between the Cα atoms of residues I122-I405 and R129-I401 in each frame of the five independent MD trajectories generated using Desmond. The TM3-TM6 distance was defined as the mean of these two pairwise distances. For each trajectory, the average TM3-TM6 distance and percentage of frames with TM3-TM6 distances greater than 12.21 Å were calculated, corresponding to the distance measured in the active-state receptor structure (PDB: 8W8S).

### 4.5 System Preparation for Free Energy Calculations

A representative frame from the MD simulation was extracted using k-means clustering of the trajectories. Water, ions, and lipids were removed. Maestro’s Induced Fit Docking (IFD) protocol with extended sampling^41–43^ was used to generate an optimized initial binding pose of reference ligand RvD5_n‑3 DPA_ in GPR101 for free energy calculations.

The Solution Builder of CHARMM-GUI’s Free Energy Calculator was used to prepare input files for relative binding free energy calculations in AMBER.^44^ For NAMD, the bilayer builder^45^ was used with a homogeneous POPC membrane. Disulfide bridges were manually assigned between residues C104 and C182 to preserve the structural integrity of the active state receptor. The complex and ligand systems were set to pH 7.4, 0.15 M NaCl was used to neutralize charges and bring the systems to physiological levels, and the TIP3P water model was used for solvation. The proteins and ligands were parametrized using the FF19SB and GAFF2 force fields, respectively.

The Membrane Builder module of CHARMM-GUI was used to prepare the bilayer system for AMBER MD simulations and subsequent MM-GBSA analyses.^46–49^ A heterogeneous lipid bilayer composed of 90% POPC and 10% cholesterol was generated to mimic the native membrane. Disulfide bridge assignment, solvation, ion addition, and parametrization were performed as previously described.

### 4.6 Relative Binding Free Energy Protocols

Amber TI networks were built with redundant edges using series-matched references (RvD5 or RvD5_n‑3 DPA_) to maximize the alchemical overlap across unsaturation patterns, in addition to assessing the transformation magnitude on convergence and statistical variance. NAMD REMD/λ-FEP calculations were run on a prioritized subset for cross-validation.

### 4.7 Thermodynamic Integration (AMBER)

Thermodynamic Integration (TI) was employed for the calculations of relative binding free energies, using the standard, concerted AMBER protocol^50^ in the AMBER 22 package, running simulations on NVIDIA RTX 2080 Ti GPUs on the University of Oslo’s Machine Learning nodes. 21–41 linearly scaled λ windows were employed for the alchemical transformations between the ligand pairs. A cutoff of 10 Å was used for the calculations of vdW interactions, the particle mesh Ewald method was used for handling long-range electrostatics, and the SCC(2) softcore potential was employed for smoothing between λ states. Hydrogen mass repartitioning was enabled and the integration time step was set to 4 fs. Minimization and 15 ps equilibration were run at each λ, before 5 ns production runs in the NPT ensemble at 310 K and 1 atm. The MBAR method was used to calculate the free energy differences between ligand pairs.

### 4.8 Free Energy Perturbation (NAMD)

Relative binding free energies were also calculated with NAMD’s free energy perturbation with λ replica exchange molecular dynamics simulation (FEP/λ-REMD) standard protocol,^51^ running on CPUs on the Saga supercomputer. A hybrid single-dual topology scheme with soft-core potential was utilized, with 32 linearly scaled λ-windows for ligand transformations, and replica-exchanges were evaluated with the conventional Metropolis-Hastings criterion. After minimization and equilibration steps, 5 ns relative FEP/ λ-REMD were performed, and the last 4 ns were used for calculation of the final relative free energies between ligand pairs.

### 4.9 MM-GBSA and Per-Residue Decomposition Analysis

Following minimization and 50 ns equilibration, three independent 50 ns production AMBER MD simulations were performed using different initial velocity seeds. Each trajectory was sampled every 100 ps, yielding 500 frames per run for the analysis. Molecular Mechanics Generalized Born Surface Area (MM-GBSA) calculations were performed using the MMPBSA.py script in AMBER.^52,53^ Per-residue free energy decomposition was conducted to identify the key contributions to ligand binding. The reported values correspond to the mean ± standard deviation across three independent simulations.

### 4.10 Molecular Dynamics and Free Energy Calculation Details

Detailed simulation system composition, membrane properties, force fields, equilibration procedures, and molecular dynamics parameters for all simulated systems (Desmond MD, AMBER TI/MM-GBSA, and NAMD FEP/λ-REMD) are summarized in Table S6.

## Supplementary Material



## Data Availability

MD trajectories, PDB and SDF files of the protein and ligands used as input for CHARMM-GUI and free-energy calculations were deposited at Zenodo (Community: LIPCHEM Molecular Modeling).

## Notes

Simulation system parameters and composition, protein backbone RMSF, ligand RMSD, residue interaction counts, TM3–TM6 activation distances, MM-GBSA per-residue energy decomposition, and relative binding free energy validation and alchemical transformation cycles (PDF)

## Abbreviations

DHA: docosahexaenoic acid
n-3 DPA: n-3 docosapentaenoic acid
EM: electron microscopy
ECL: extracellular loop
FEP: free energy perturbation
GPCR: G protein-coupled receptor
IFD: induced fit docking
MD: molecular dynamics
MM: molecular modeling
MMGBSA: molecular mechanics generalized Born surface area
MMPBSA: molecular mechanics Poisson–Boltzmann surface area
NPT: constant particles, pressure, and temperature ensemble
OPM: orientation of proteins in membranes
PDB: Protein Data Bank
PGDH: prostaglandin dehydrogenase
POPC: 1-palmitoyl-2-oleoyl-sn-glycero-3-phosphocholine
RBFE: relative binding free energies
REMD: replica exchange molecular dynamics
RMSD: root-mean-square deviation
RvD5: resolvin D5
RvD5_n‑3 DPA_: resolvin D5 n-3 docosapentaenoic acid
SEM: standard error of the mean
SPM: specialized pro-resolving mediator
TI: thermodynamic integration
TM: transmembrane
