# Supplementary material for: Molecular Dynamics and Free Energy Calculations Predict Binding Mode and Affinity Determinants of Specialized Pro-Resolving Mediators at GPR101
Source: ACS Omega. 2026 Jul 12;11(29):43166–76. doi: 10.1021/acsomega.5c13583 (PMC13425295; doi:10.1021/acsomega.5c13583)
Supplement: Supplementary file 1 [file ao5c13583_si_001.pdf]

Supporting information to Molecular dynamics and free energy calculations predict binding mode and affinity determinants of specialized pro-resolving mediators at GPR101

Daniel Haga Hasselstrøm[1], Majd Awad[1], Trond Vidar Hansen[1], Osman Gani[1].

[1] Department of Pharmacy, Section for Pharmaceutical Chemistry, University of Oslo, P.O. Box 1068, 0316 Oslo, Norway.

Table of Contents

Table S1. Protein backbone RMSF (Å) from TM regions, from five independent 1 μs runs of MD simulation of $RvD5_{n\text{-}3\ DPA}$ in GPR101 2
Table S2. Mean ligand RMSD (Ångström) from five independent 1 μs runs of MD simulation of $RvD5_{n\text{-}3\ DPA}$ in GPR101..2
Figure S1. Ligand RMSD (Ångström) from five independent 1 μs runs of molecular dynamics simulations of $RvD_{5n\text{-}3\ DPA}$ in the orthosteric binding site of GPR101..3
Table S3. Normalized interaction counts with residues from five independent 1 μs molecular dynamics simulation runs of $RvD5_{n\text{-}3\ DPA}$ in the GPR101 orthosteric site.. 5
Table S4. TM3–TM6 distances calculated from five replicate molecular dynamics simulations of $RvD5_{n\text{-}3\ DPA}$ in the orthosteric binding site of GPR101..6
Figure S2. Mean TM3-TM6 distance for GPR101 and $RvD5_{n\text{-}3\ DPA}$ across five independent 1 μs MD trajectories. 6
Figure S3. Bar chart showing the percentage of frames in the active-like state (TM3–TM6 distance ≤ 12.21 Å) for GPR101 and $RvD5_{n\text{-}3\ DPA}$ across five independent MD simulation replicates..........................7
Table S5. MM-GBSA per residue decomposition (kcal/mol) of $RvD5_{n\text{-}3\ DPA}$ from three independent 50 ns MD simulation runs using Amber..8
Figure S4. Correlation plot between AMBER TI and NAMD FEP/λ-REMD relative binding free energies (ΔΔG). 9
Figure S5. Binding free energy differences (ΔΔG) for alchemical transformations between $RvD5_{n\text{-}3DPA}$, RvD5 and AT-RvD5, computed using AMBER Thermodynamic Integration. 10
Figure S6. Binding free energy differences (ΔΔG) for alchemical transformations between $RvD5_{n\text{-}3DPA}$, RvD5 and 17-oxo-RvD5, computed using AMBER Thermodynamic Integration. 11
Figure S7. Binding free energy differences (ΔΔG) for alchemical transformations between $RvD5_{n\text{-}3\ DPA}$, RvD5 and 17(S)-Me-RvD5, computed using AMBER Thermodynamic Integration..........................12
Figure S8. Binding free energy differences (ΔΔG) for alchemical transformations between $RvD5_{n\text{-}3DPA}$, RvD5 and 17(R)-Me-RvD5, computed using AMBER Thermodynamic Integration... 13
Table S6. Simulation system composition, membrane properties, and molecular dynamics parameters for all simulated systems..2

Run	Mean protein TM backbone RMSF ± SD (Å)
Run 1	1.45 ± 0.83
Run 2	1.30 ± 0.91
Run 3	1.19 ± 0.79
Run 4	1.27 ± 0.83
Run 5	1.41 ± 0.87
Mean of runs	**1.33 ± 0.85**

Table S1. Protein backbone RMSF (Å) from TM regions, from five independent 1 μs runs of MD simulation of RvD5$_{n\text{-}3\ DPA}$ in GPR101

Run	Mean ligand RMSD ± SD (Å)
Run 1	2.19 ± 0.53
Run 2	1.86 ± 0.49
Run 3	1.94 ± 0.35
Run 4	1.60 ± 0.23
Run 5	1.91 ± 0.32
Mean of runs	**1.90 ± 0.44**

Table S2. Mean ligand RMSD (Ångström) from five independent 1 μs runs of MD simulation of RvD5$_{n\text{-}3\ DPA}$ in GPR101.

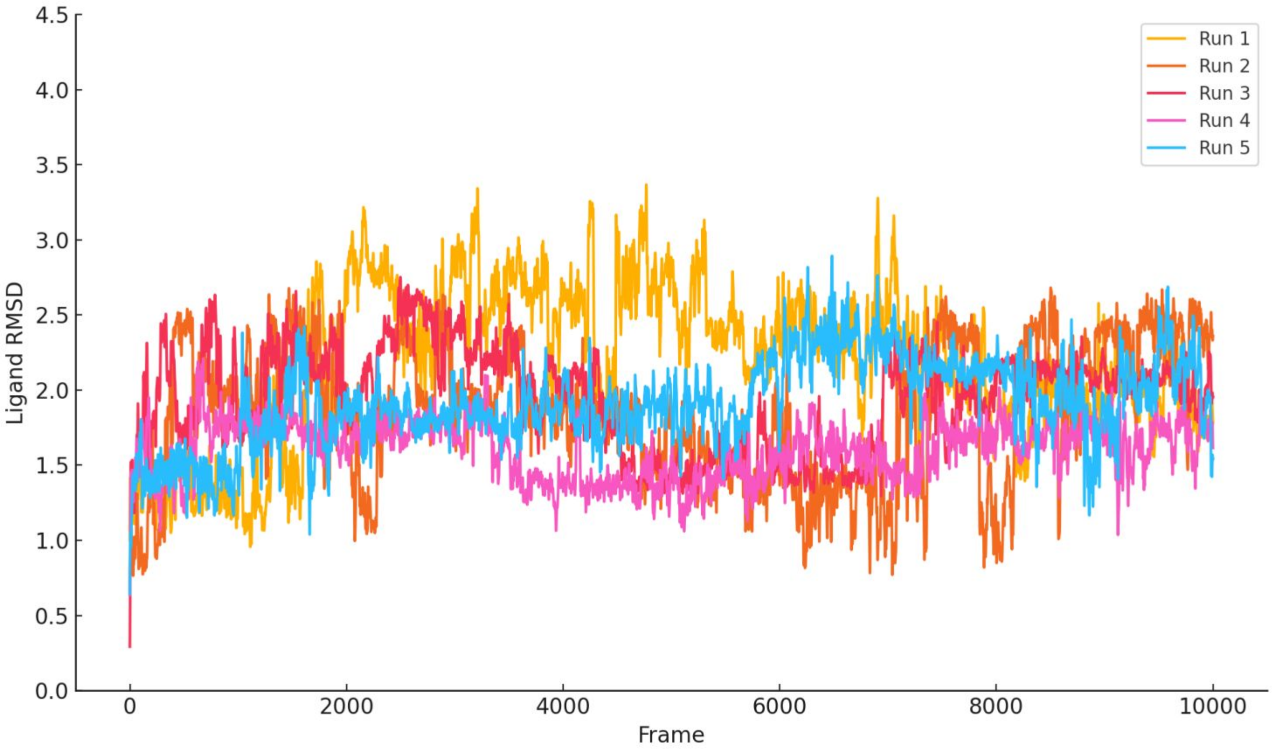

Figure S1. Ligand RMSD (Ångström) from five independent 1 μs runs of molecular dynamics simulations of RvD$_{5n\text{-}3\ DPA}$ in the orthosteric binding site of GPR101.

Residue/Run	Run1	Run2	Run3	Run4	Run5	Mean	Std. Dev.
R34	0.312	0.000	0.000	0.001	0.002	0.063	0.139
V84_nonpolar	0.062	0.107	0.204	0.089	0.039	0.100	0.064
W87_nonpolar	0.179	0.044	0.082	0.024	0.000	0.066	0.070
V88_nonpolar	0.091	0.072	0.102	0.289	0.201	0.151	0.092
L99_nonpolar	0.000	0.020	0.075	0.000	0.000	0.019	0.032
L107_nonpolar	0.147	0.094	0.074	0.051	0.023	0.078	0.047
V108_nonpolar	0.015	0.027	0.034	0.040	0.045	0.032	0.012
T111	0.074	0.023	0.018	0.010	0.000	0.025	0.029
F175	0.022	0.139	0.005	0.000	0.089	0.051	0.061
D176	0.665	0.672	0.384	0.186	0.433	0.468	0.205
R178	0.819	0.449	0.344	0.944	0.276	0.566	0.297
N179	0.636	0.244	0.157	0.045	0.112	0.239	0.234
L181_nonpolar	0.191	0.172	0.298	0.507	0.284	0.290	0.133
C182	0.917	0.947	0.867	0.866	0.849	0.889	0.041
C182_nonpolar	0.000	0.000	0.000	0.042	0.0	0.008	0.019
S183	0.460	0.211	0.390	0.161	0.383	0.321	0.128
M184	0.507	1.037	0.760	0.735	0.694	0.747	0.190
W186	0.359	0.115	0.477	0.066	0.183	0.240	0.173
W186_nonpolar	0.058	0.084	0.024	0.254	0.186	0.121	0.096
G187	0.116	0.293	0.331	0.003	0.274	0.203	0.139
Y415	0.429	0.790	0.499	0.450	0.102	0.454	0.245
Y415_nonpolar	0.720	0.612	0.602	0.834	0.799	0.713	0.106
L418_nonpolar	0.272	0.317	0.047	0.486	0.292	0.283	0.157

A422	0.238	0.342	0.363	0.027	0.230	0.240	0.133
A422_nonpolar	0.045	0.034	0.094	0.007	0.036	0.043	0.032
V425	0.125	0.123	0.198	0.000	0.050	0.099	0.076
D426	0.179	0.394	0.436	0.000	0.163	0.234	0.180
V427	0.234	0.202	0.341	0.052	0.094	0.185	0.115
V427_nonpolar	0.074	0.082	0.126	0.166	0.116	0.113	0.037
E428	0.597	0.546	1.393	0.261	1.247	0.809	0.487
T429	0.355	0.044	0.007	1.248	0.173	0.365	0.512
V431	0.181	0.011	0.001	0.713	0.03	0.187	0.303
V431_nonpolar	0.000	0.000	0.001	0.082	0.000	0.017	0.037
N433	0.358	0.130	0.195	0.842	0.057	0.316	0.314
I436_nonpolar	0.067	0.116	0.173	0.048	0.163	0.113	0.056
I439_nonpolar	0.101	0.005	0.005	0.103	0.145	0.072	0.063
I440_nonpolar	0.428	0.434	0.398	0.100	0.156	0.303	0.162
F443_nonpolar	0.182	0.362	0.369	0.198	0.164	0.255	0.102
F444_nonpolar	0.100	0.071	0.085	0.120	0.150	0.105	0.031

Table S3. Normalized interaction counts with residues from five independent 1 μs molecular dynamics simulation runs of RvD5$_{n\text{-}3\ DPA}$ in the GPR101 orthosteric site. Per-run values are shown along with the mean and standard deviation across the simulations. Interactions are categorized as polar or nonpolar based on residue-specific interaction characteristics.

Run	Mean TM3-TM6 distance ± SD (Å)	Fraction of frames over 12.21 Å (%)
Run 1	11.87 ± 0.85	40.3 %
Run 2	11.86 ± 0.91	41.4 %
Run 3	11.30 ± 0.79	12.2 %
Run 4	11.87 ± 0.74	29.3 %
Run 5	10.92 ± 0.73	5.5 %
Mean of runs	**11.56 ± 0.44**	**25.8 ± 16.3 %**

Table S4. TM3–TM6 distances calculated from five replicate molecular dynamics simulations of RvD5$_{n\text{-}3\ DPA}$ *in the orthosteric binding site of GPR101.*

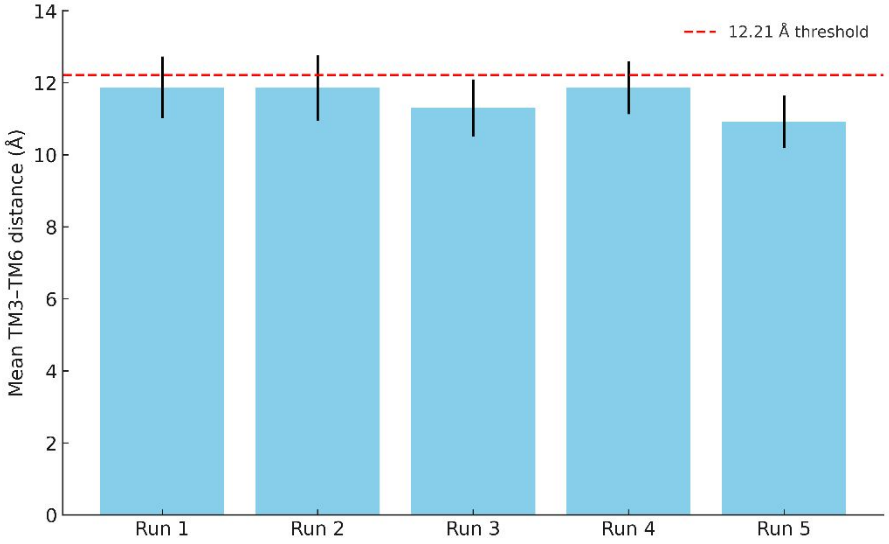

Figure S2. Mean TM3-TM6 distance for GPR101 and RvD5$_{n\text{-}3\ DPA}$ *across five independent 1 µs MD trajectories. Error bars represent the standard deviation. The horizontal red dashed line marks the active-like threshold (12.21 Å).*

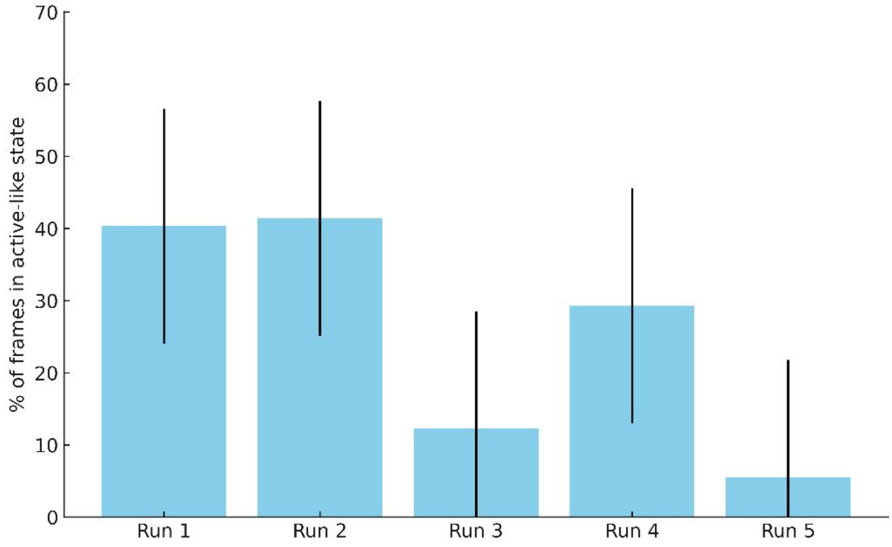

Figure S3. Bar chart showing the percentage of frames in the active-like state (TM3–TM6 distance ≤ 12.21 Å) for GPR101 and RvD5$_{n\text{-}3\ DPA}$ across five independent MD simulation replicates. The standard deviations are shown as black error bars.

Mean (kcal/mol)	Std. Dev. (kcal/mol)	Residue
-3.65	0.04	L181
-2.57	0.09	C182
-2.09	0.12	S183
-1.63	0.94	N179
-1.61	0.2	L418
-1.51	0.46	W186
-1.42	0.06	I440
-1.41	0.16	M184
-1.28	0.08	V108
-1.26	0.14	V427
-1.13	0.06	V84
-1.09	0.09	L107
-1.02	0.08	W87
-0.96	0.09	V88
-0.88	0.34	I436
-0.86	0.08	T111
-0.73	0.86	R34

-0.69	0.24	Y415
-0.54	0.01	C104
-0.47	0.12	F443
-0.45	0.08	A422
-0.45	0.06	L83
-0.44	0.16	R178
-0.44	0.06	I439
-0.43	0.18	A419
-0.43	0.07	F444
-0.41	0.25	Q433
-0.36	0.06	T91
-0.33	0.02	P98
-0.33	0.03	F175
-0.26	0.02	L38
-0.26	0.03	A173
-0.24	0.02	L110
-0.2	0.02	L421
-0.19	0.04	F103
-0.17	0.01	Q80
-0.15	0.02	F417
-0.14	0.08	I185
-0.14	0.03	L442
-0.13	0.01	A180

Table S5. MM-GBSA per residue decomposition (kcal/mol) of RvD5$_{n\text{-}3\ DPA}$ from three independent 50 ns MD simulation runs using Amber.

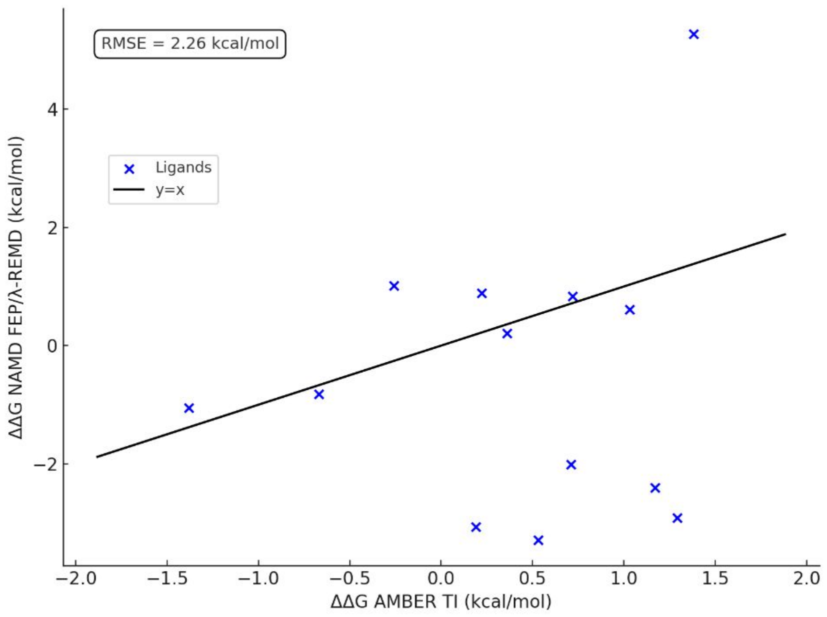

Figure S4. Correlation plot between AMBER TI and NAMD FEP/λ-REMD relative binding free energies (ΔΔG). The black line represents perfect correlation (y = x), and the RMSE is shown in the top-left box.

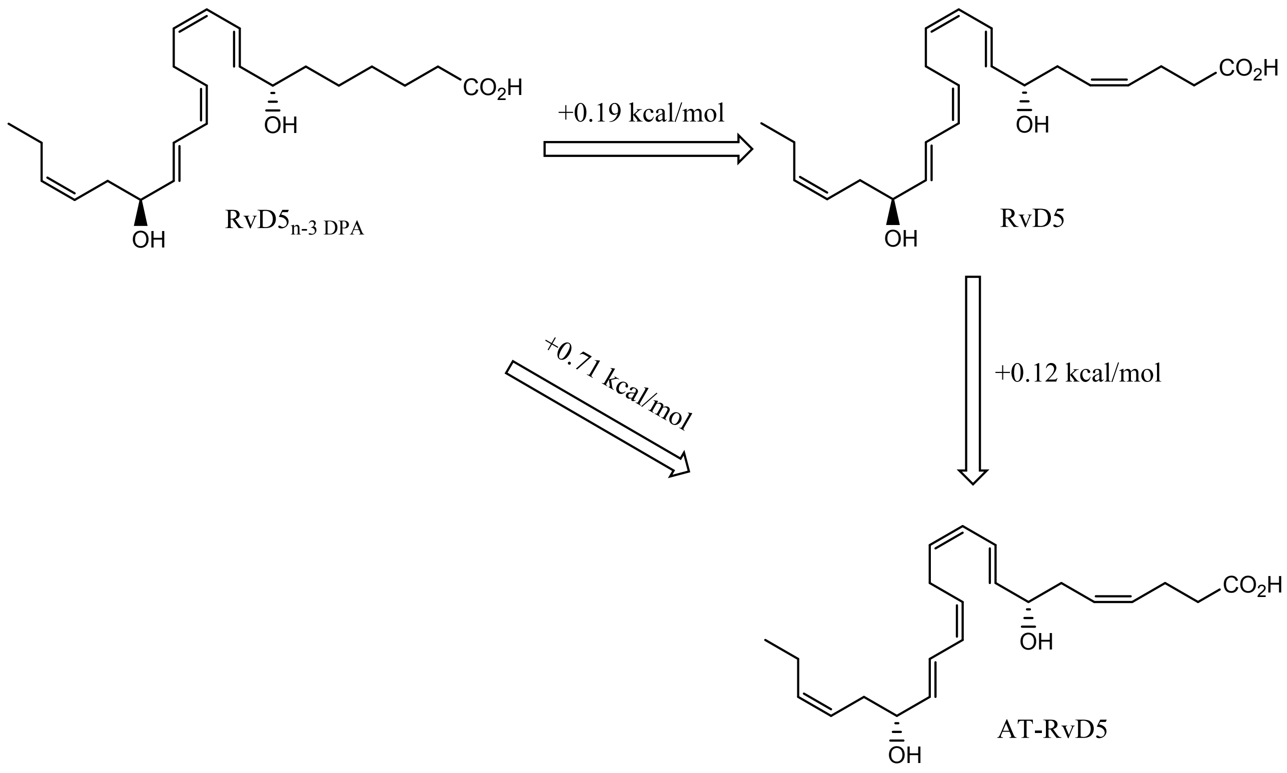

Figure S5. Binding free energy differences (ΔΔG) for alchemical transformations between RvD5$_{n\text{-}3DPA}$, RvD5 and AT-RvD5, computed using AMBER Thermodynamic Integration. The calculated cycle closure error was -0.40 kcal/mol.

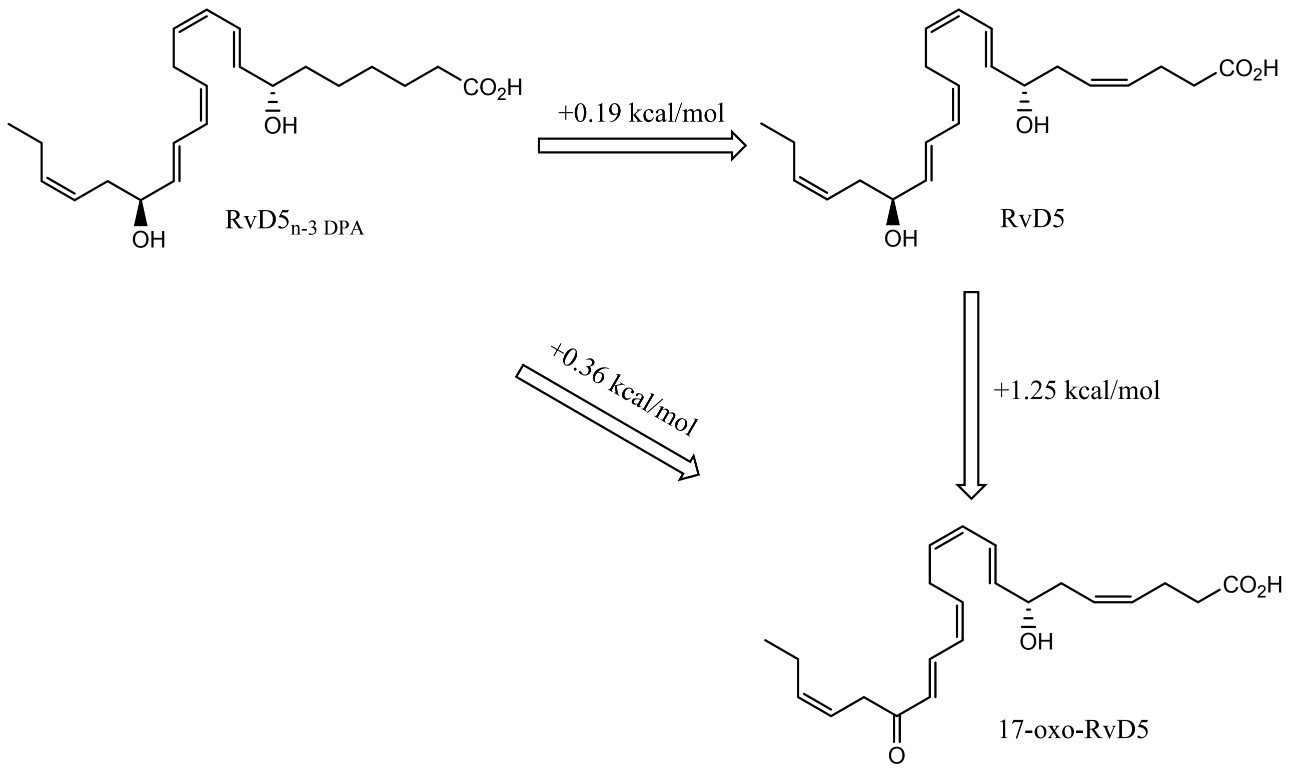

Figure S6. Binding free energy differences (ΔΔG) for alchemical transformations between RvD5$_{n\text{-}3DPA}$, RvD5 and 17-oxo-RvD5, computed using AMBER Thermodynamic Integration. The calculated cycle closure error was +1.08 kcal/mol.

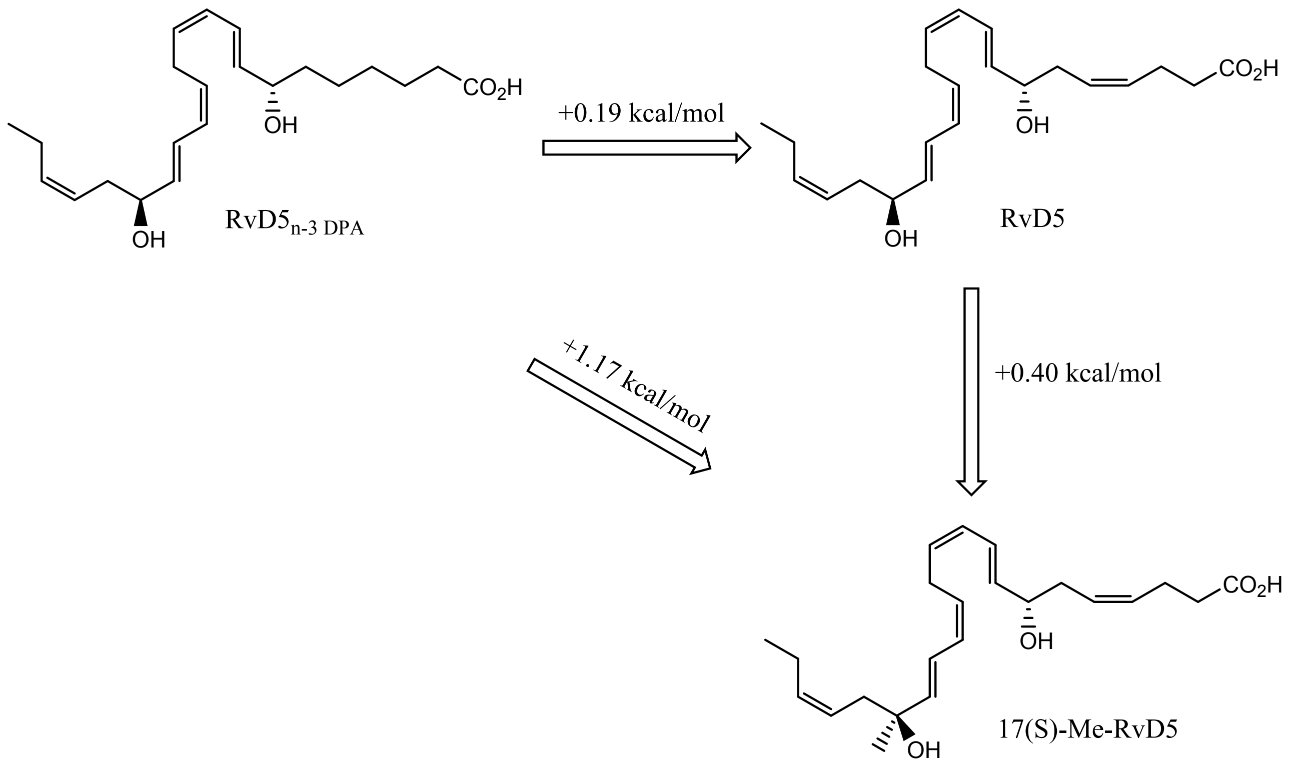

Figure S7. Binding free energy differences (ΔΔG) for alchemical transformations between RvD5$_{n\text{-}3\ DPA}$, RvD5 and 17(S)-Me-RvD5, computed using AMBER Thermodynamic Integration. The calculated cycle closure error was -0.58 kcal/mol.

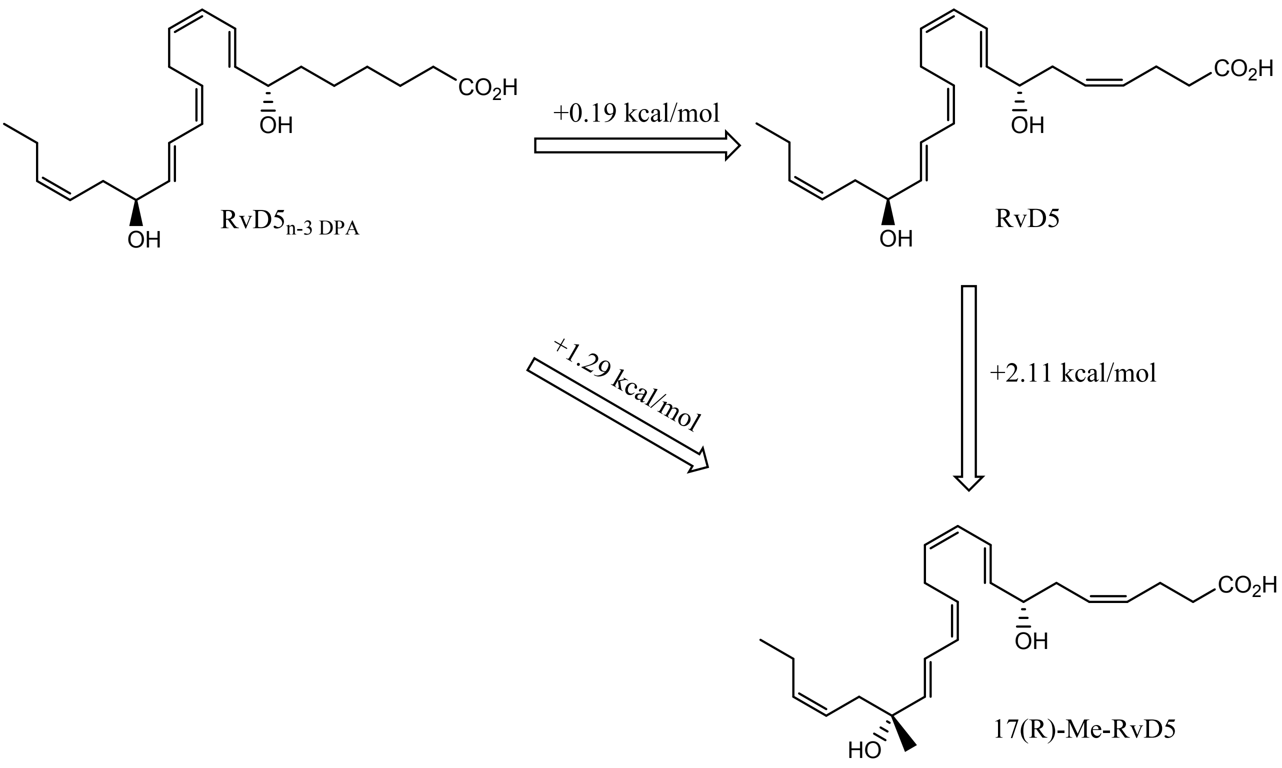

Figure S8. Binding free energy differences (ΔΔG) for alchemical transformations between RvD5$_{n\text{-}3DPA}$, RvD5 and 17(R)-Me-RvD5, computed using AMBER Thermodynamic Integration. The calculated cycle closure error was +1.01 kcal/mol.

Category	Desmond MD	AMBER MM-GBSA	AMBER TI	NAMD FEP
Purpose	Binding mode stability and residue interaction analysis	Per-residue energy decomposition (MM-GBSA)	Relative binding free energy (Thermodynamic Integration)	Relative binding free energy (FEP / λ-REMD)
Force Field (Protein)	OPLS4	ff19SB	ff19SB	CHARMM36m
Force Field (Ligand)	OPLS4	GAFF2	GAFF2	CGenFF
Charge Model	CM1A / OPLS4 default	AM1-BCC	AM1-BCC	CGenFF
Lipid Composition	POPC bilayer (110 lipids)	POPC bilayer (91 lipids) + CHOL (10)	Membrane-free	POPC (99 lipids)
Water Model	TIP3P	TIP3P	TIP3P	TIP3P
Ion Concentration	0.15 M NaCl	0.15 M NaCl	0.15 M NaCl	0.15 M NaCl
Total Atoms	~48,800	~51,000	~78,000	~44,000
Periodic Box (Å)	66.7 × 69.8 × 101.6	63.9 × 63.9 × 100.9	86.0 × 86.0 × 86.0	67.2 × 67.2 × 104.1
Area per lipid (Å^2)	84.6	80.9	-	91.2
Ensemble	NPT	NPT	NVT	NPT
Temperature	310 K	310 K	310 K	310 K
Thermostat	Nose–Hoover chain (τ = 1.0 ps)	Langevin (γ = 1.0 ps^{-1})	Langevin	Langevin (damping = 1.0 ps^{-1})
Barostat	Martyna–Tobias–Klein (τ = 2.0 ps)	Monte Carlo (1 atm)	None	Langevin piston
Production timestep	2 fs (bonded/short-range), 6 fs (long-range, RESPA)	4 fs (HMR, SHAKE on H-bonds)	4 fs (HMR, SHAKE on H-bonds)	2 fs
vdW Cutoff	9 Å	10 Å	10 Å	12 Å (switching on; switchdist 10 Å; pairlistdist 13.5 Å)
Electrostatics	PME	PME	PME	PME
Electrostatics cutoff	9 Å	10 Å	10 Å	12 Å

Constraints	SHAKE (bonds involving H)	SHAKE (bonds involving H)	SHAKE (bonds involving H)	Rigid bonds for water
Minimization	Desmond Minimax minimization	15,000 steps (Steepest descent for first 3,500, then conjugate gradient)	1000 steps (Conjugate gradient)	No explicit energy minimization; system relaxation via staged equilibration
Equilibration	Multi-stage relaxation/equilibration (multisim stages 2–5): Stage 2 Brownian dynamics NVT at 10 K with solute heavy-atom restraints (100 ps); Stages 3–4 at 100 K with H2O barrier, membrane restrained in z and protein restrained (Brownian NPT / NPgT); Stage 5 NPgT heating 100→300 K with gradual release of restraints.	6-stage equilibration at 310 K (5.375 ns total; timestep ramp 1→2 fs). Dihedral restraints were applied.	5 ps NPT (Langevin, no restraints)	6-stage equilibration at 310 K (1.875 ns total; timestep ramp 1→2 fs). Langevin piston enabled from stage 3 onward. Colvars and dihedral restraints were applied.
Hardware	GPU-accelerated (DESMOND_GPGPU)	GPU-accelerated (pmemd.cuda)	GPU-accelerated (pmemd.cuda)	CPU

Table S6. Simulation system composition, membrane properties, and molecular dynamics parameters for all simulated systems
